# Supplementary material for: Seismicity at the Castor gas reservoir driven by pore pressure diffusion and asperities loading
Source: Nat Commun. 2021 Aug 10;12:4783. doi: 10.1038/s41467-021-24949-1 (PMC8355105; doi:10.1038/s41467-021-24949-1)
Supplement: Supplementary file 1 — Supplementary Information [file 41467_2021_24949_MOESM1_ESM.pdf]

Supplementary Material

Seismicity at the Castor gas reservoir driven by pore pressure diffusion and asperities loading

Simone Cesca[1,*], Daniel Stich[2,3], Francesco Grigoli[4,5], Alessandro Vuan[6], José Ángel López-Comino[2,3,7], Peter Niemz[1,7], Estefanía Blanch[8,9], Torsten Dahm[1], William L. Ellsworth[10]

1. GFZ German Research Centre for Geosciences Potsdam, Germany
2. Instituto Andaluz de Geofísica, Universidad de Granada, Granada, Spain.
3. Departamento de Física Teórica y del Cosmos, Universidad de Granada, Granada, Spain
4. Department of Earth Sciences, University of Pisa, Italy
5. Swiss Seismological Service, ETH Zurich, Zurich, Switzerland
6. National Institute of Oceanography and Applied Geophysics - OGS, Trieste, Italy
7. Institute of Geosciences, University of Potsdam, Potsdam-Golm, Germany.
8. Departament de Física-EPSEB, UPC Barcelona Tech, Barcelona, Spain
9. Observatori de l’Ebre (OE), CSIC—Universitat Ramon Llull, Roquetes, Spain
10. Department of Geophysics, Stanford University, California, United States

* Corresponding author: Dr. Simone Cesca, GFZ German Research Centre for Geosciences Potsdam, Germany, simone.cesca@gfz-potsdam.de

Supplementary Note 1, Depth of injection-induced earthquakes

We recompiled a list of the earthquakes with magnitude 5.0 or larger and occurring since 1.1.2000, which have been related to fluid injection and/or extraction operations (Supplementary Table 1). The original list, built upon a broad induced seismicity catalog, has been extended for a few more recent earthquakes. Note that we excluded a few events from the original catalog, for which an induced origin is not verified, such as those listed in Iran and Nepal. We report for completeness two disputed cases, those in Lorca (Spain) and Emilia Romagna (Italy). Source depths, as reported by dedicated seismological studies[1,2,3], are in the range 5.2±1.4 km and never exceed 8.3 km.

Name	Operations	Date	Mag	Depth
Salton Sea, California (USA)	EGS circulation	31.08.2005	5.1[1], 4.6	4.0
Lorca (Spain)	*Groundwater extraction*	*11.05.2011*	*5.1*[1]	*4.6*
Emilia (Italy)	*Oil and gas, Wastewater injection*	*20.05.2012*	*5.9*[1]	*7.0*[2]
Prague, Oklahoma (USA)	Wastewater injection	06.11.2011	5.7[1]	5.2
Raton Basin, Colorado and New Mexico (USA)	Wastewater injection	23.08.2011	5.3[1]	4.0
Fairview, Oklahoma (USA)	Wastewater injection	13.02.2016	5.1[1]	8.3
Pohang (South Korea)	EGS Stimulation	15.11.2017	5.5[3]	4.0
Pawnee, Oklahoma (USA)	Wastewater injection	13.02.2016	5.8	5.6
Cushing, Oklahoma (USA)	Wastewater injection	07.11.2016	5.0	4.4

Supplementary Tab. 1: List of large, recent earthquakes associated to fluid injection, extraction and/or storage.

The table reports location name, type of operation, date of occurrence, magnitude estimates and depth (in km). Disputed cases of induced seismicity are in italic. Magnitude and depths are according to the USGS seismic catalog, except for those cases where a specific reference is provided.

Supplementary Note 2, Seismic dataset used

We use seismic data from the following networks (Suppl. Fig. 1): ES – Spanish Digital Seismic Network[4], CA – Catalan Seismic Network[5], WM – Western Mediterranean Seismic Network[6], EB - Ebre Observatory Regional Seismic Network, IB - IberArray[7], GR – German Regional Seismic Network[8] and ILAR array (Eielson, Alaska, US). Seismic data and metadata have been accessed using IRIS, Geofon, Orfeus and (ICGC) web services, or alternatively kindly provided by the Ebre Observatory and IGN.

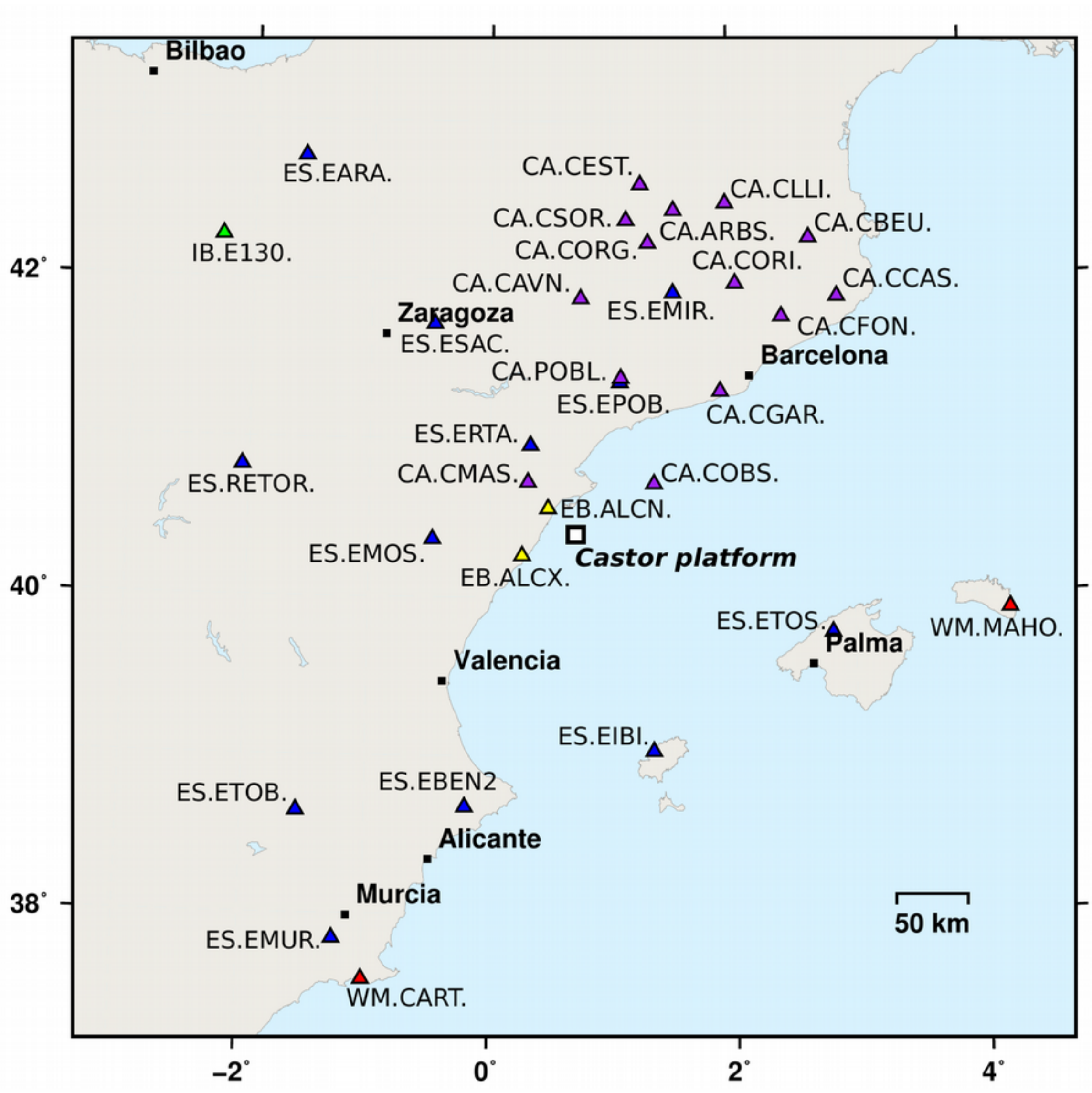

Supplementary Fig. 1: Map of the 31 permanent and temporal seismic stations used in this work.

Station locations are marked by triangles, with colors correspond to different networks: ES – Spanish Digital Seismic Network[4], blue triangles, CA – Catalan Seismic Network[5], purple triangles, WM – Western Mediterranean Seismic Network[6], red triangles, EB - Ebre Observatory Regional Seismic Network, yellow triangles and IB - IberArray[7], green triangles. All stations are instrumented with broadband seismometers, except EB.ALCN and EB.ALCX, which are instrumented with short period sensors. A white square denote the location of the Castor platform. Major cities are listed by name and their locations indicated by small black squares.

Supplementary Note 3, Crustal velocity models

A variety of crustal velocity models have been used for hypocentral location and moment tensor determination for the Castor seismicity[9,10]. In this study, we consider the following 1D models (Suppl. Fig. 2). Model I, proposed for the Iberian Peninsula[11], is currently used at IGN for standard location procedures. Model V is a local 1D model extracted[10] from a 2D model[12], based on the processing of a seismic transect across the Valencia Through. Model G is a 1D model[10] extracted for the Castor location out of a 3D model, proposed from a surface-wave ambient noise tomography[10].

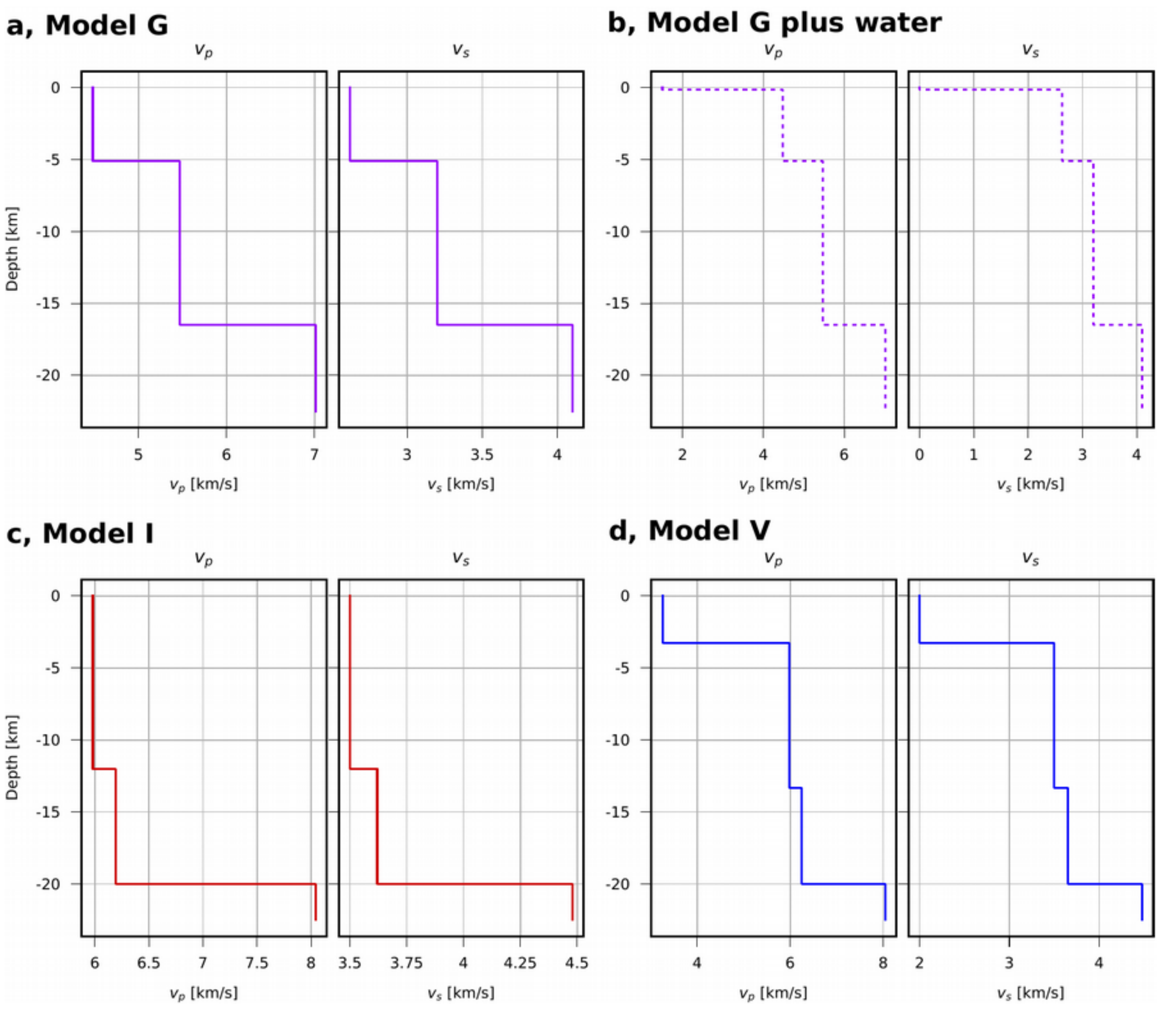

Supplementary Fig. 2: Velocity models used in this study.

a, Model G[10], plotted with purple solid lines). **b,** Model G with a shallow water layer, with purple dashed lines. **c,** Model I[11], with red solid lines. **d,** Model V[12], with blue solid lines. For each model we provide profiles for P and S waves down to 22.5 km, including the crust-mantle boundary.

Supplementary Note 4, Seismic catalags

We provide three seismic catalogs as supplementary material:

a. Template matching catalog, including 3,437 events (Supplementary Dataset 1).

b. Relative location catalog based on waveform correlation, including 51 events (Supplementary Dataset 2).

c. Relocated catalog based on tS-tP and distance geometry technique, including 408 events (Supplementary Dataset 3).

Note that event naming and timing may differ from catalog to catalog.

Details of moment tensor and directivity inversion results, providing advanced catalogs for selected largest earthquakes, are provided in the dedicated sections of this document.

Supplementary Note 5, Template matching and statistical properties of the sequence

Template matching is used to detect weaker seismic signals, similar to those of reference events in the available seismic catalogs, in order to enhance the catalog including weaker events. The application to the Castor sequence led to the compilation of a catalog of 3,437 earthquakes. We complement here our manuscript by showing example of detections of weak events by template matching (Suppl. Figs. 3, 4, 5) and showing the temporal evolution of the extended seismic catalog, in comparison to the Ebro catalog (Suppl. Fig. 6).

Statistical analysis of the magnitude distribution of the extended catalog, e.g. by means of b-values, require the estimation of magnitudes for new detected events. This is achieved knowing the magnitude of the master events, and the amplitude scaling among master and new detected events. We noted, however, a discrepancy among magnitude in the original IGN and Ebro catalogs, which can be attributed to different magnitude estimation approaches[13]. Using the Ebro catalog event, we confirmed a substantial decrease of the b-value after shut-in (Suppl. Fig. 7), thus confirming previous similar findings[9]. Using the IGN catalog as reference, we also depict a decrease of the b-value, but b-values in both phases are underestimated.

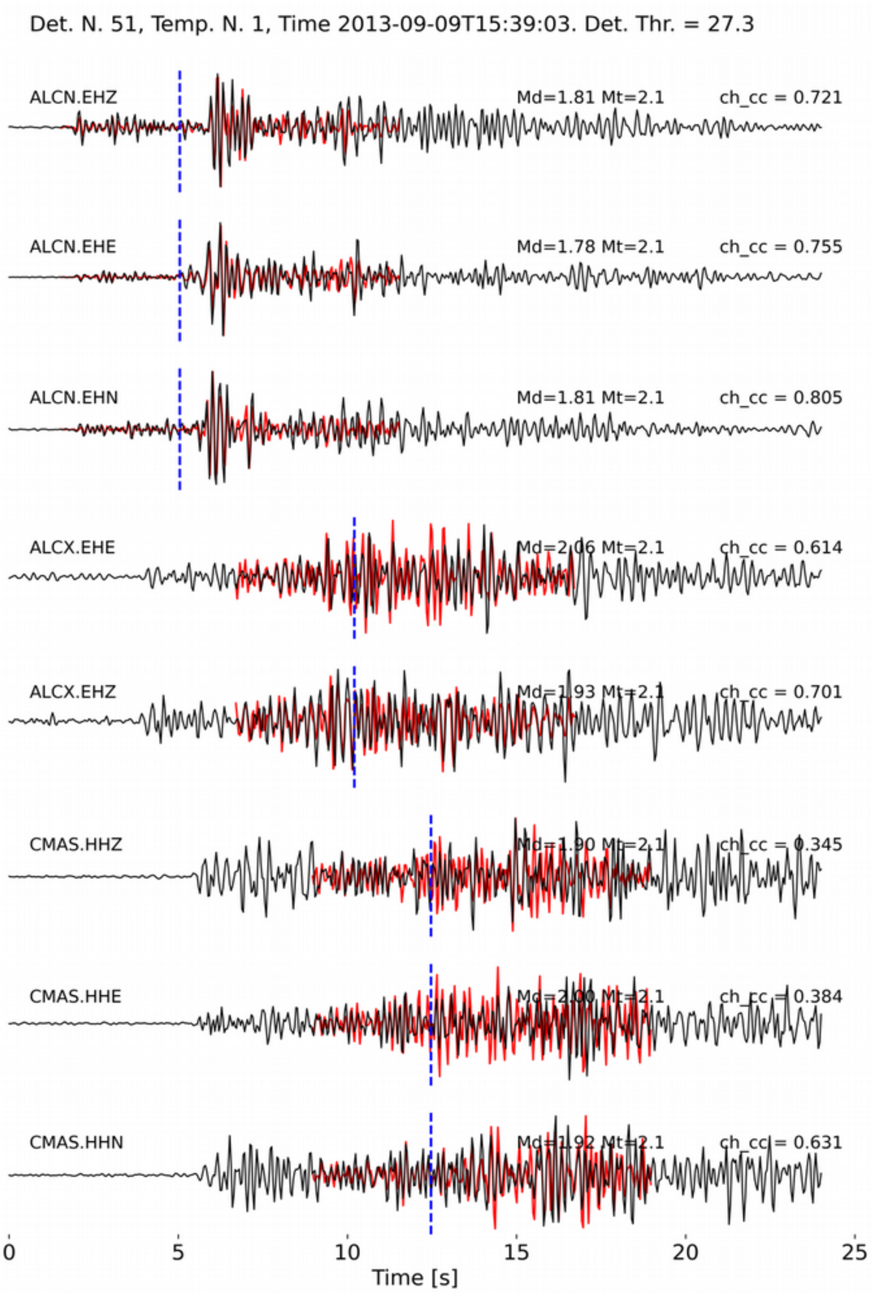

Supplementary Fig. 3: Examples of detections using template matching, for a magnitude M1.8 earthquake.

The templates (red traces) are superimposed on the continuous data (black traces). Next to each trace we report the station and channel codes (left), single-channel estimated magnitude (Md, middle) and the cross-correlation between the template and the new detection (right). The top title lists the magnitude of the template, the time of the detection, and the threshold. The dashed blue vertical lines represent the theoretical S-wave arrival used as a reference to trim the template waveforms.

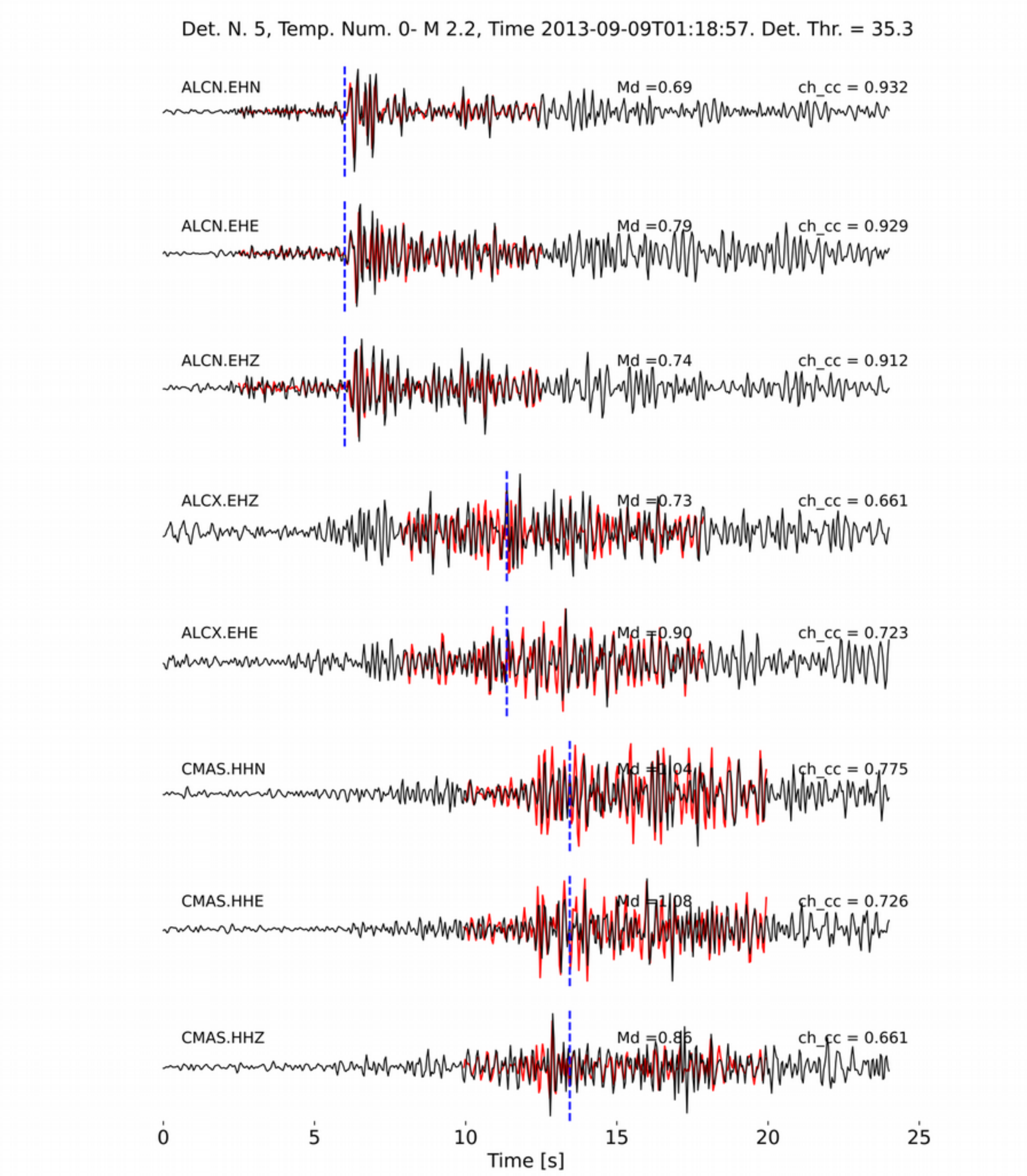

Supplementary Fig. 4: Examples of detections using template matching, for a magnitude M0.8 earthquake.

The templates (red traces) are superimposed on the continuous data (black traces). Next to each trace we report the station and channel codes (left), single-channel estimated magnitude (Md, middle) and the cross-correlation between the template and the new detection (right). The top title lists the magnitude of the template, the time of the detection, and the threshold. The dashed blue vertical lines represent the theoretical S-wave arrival used as a reference to trim the template waveforms.

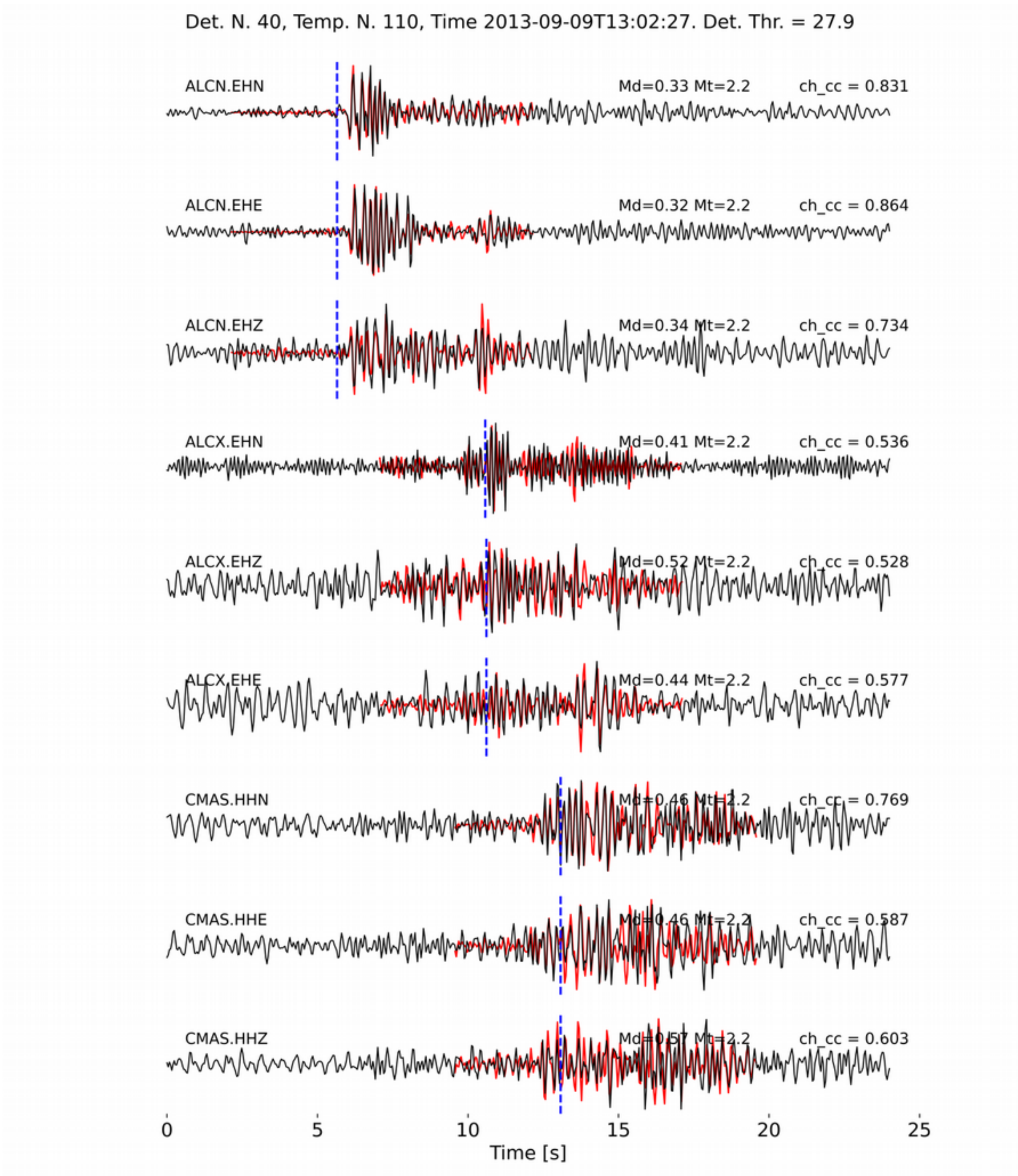

Supplementary Fig. 5: Examples of detections using template matching, for a magnitude M0.4 earthquake.

The templates (red traces) are superimposed on the continuous data (black traces). Next to each trace we report the station and channel codes (left), single-channel estimated magnitude (Md, middle) and the cross-correlation between the template and the new detection (right). The top title lists the magnitude of the template, the time of the detection, and the threshold. The dashed blue vertical lines represent the theoretical S-wave arrival used as a reference to trim the template waveforms.

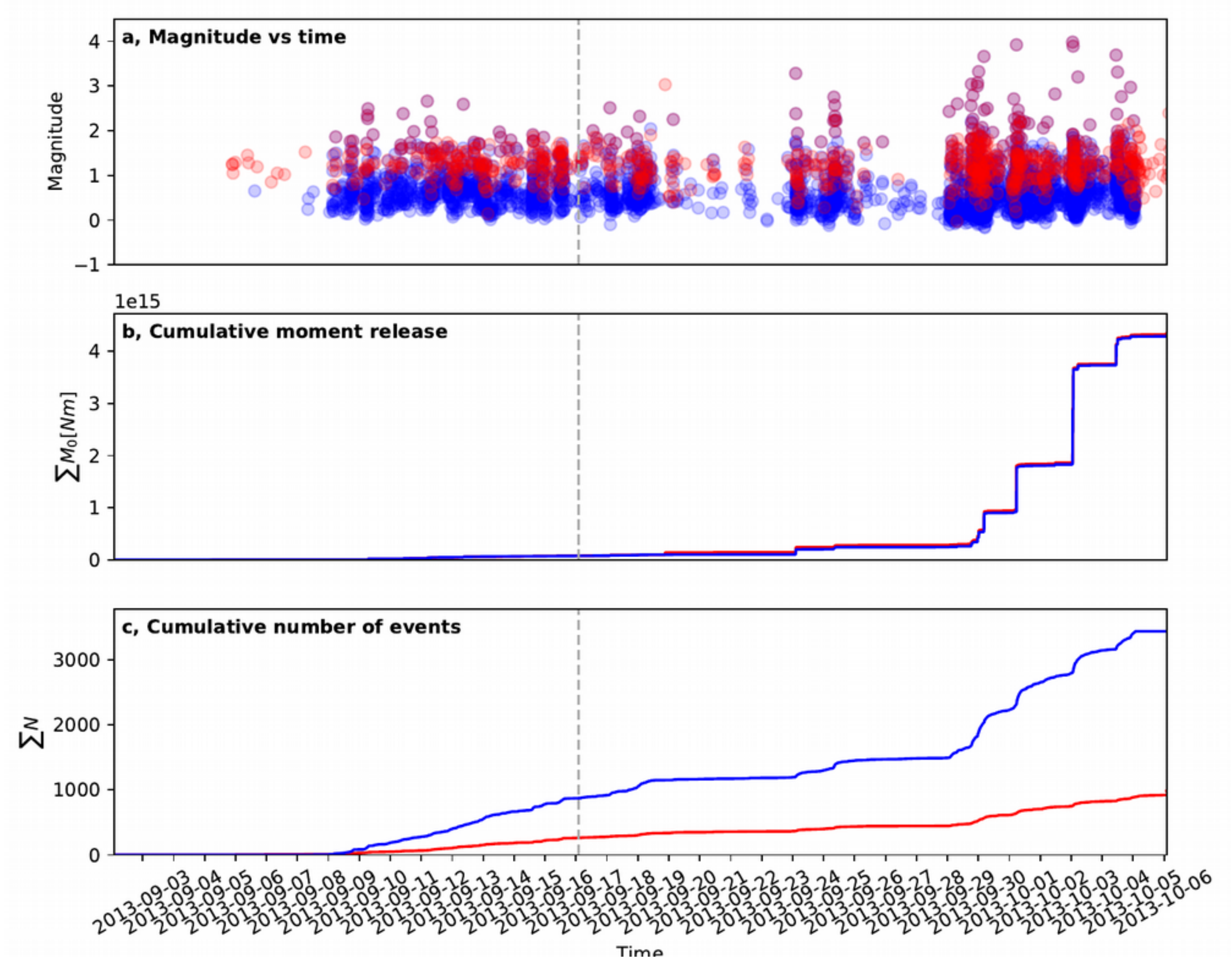

Supplementary Fig. 6: Comparison of the original Ebro catalog with the extended catalog after using template matching.

a, Temporal evolution of the magnitudes, **b,** the cumulative scalar moment release and **c,** the cumulative number of events. Red color is used for the Ebro catalog, blue for our extended catalog. A dashed gray line marks the time of injection stop.

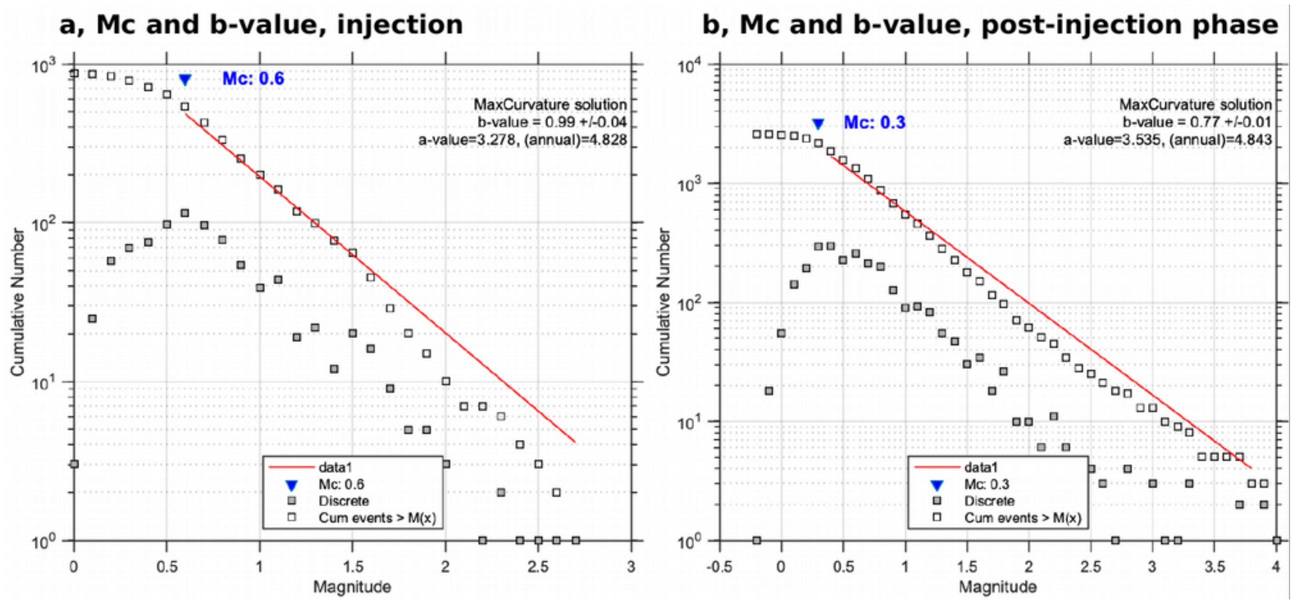

Supplementary Fig. 7: Temporal variation of b-values and magnitude of completeness.

Mc and b-value estimates are shown for **a,** the injection and **b,** post-injection phases. The magnitude of completeness has been derived using the maximum curvature approach[14]. We considered here the extended seismic catalog after applying template matching and with magnitudes based on the Ebro catalog.

Supplementary Note 6, Seismicity relocation

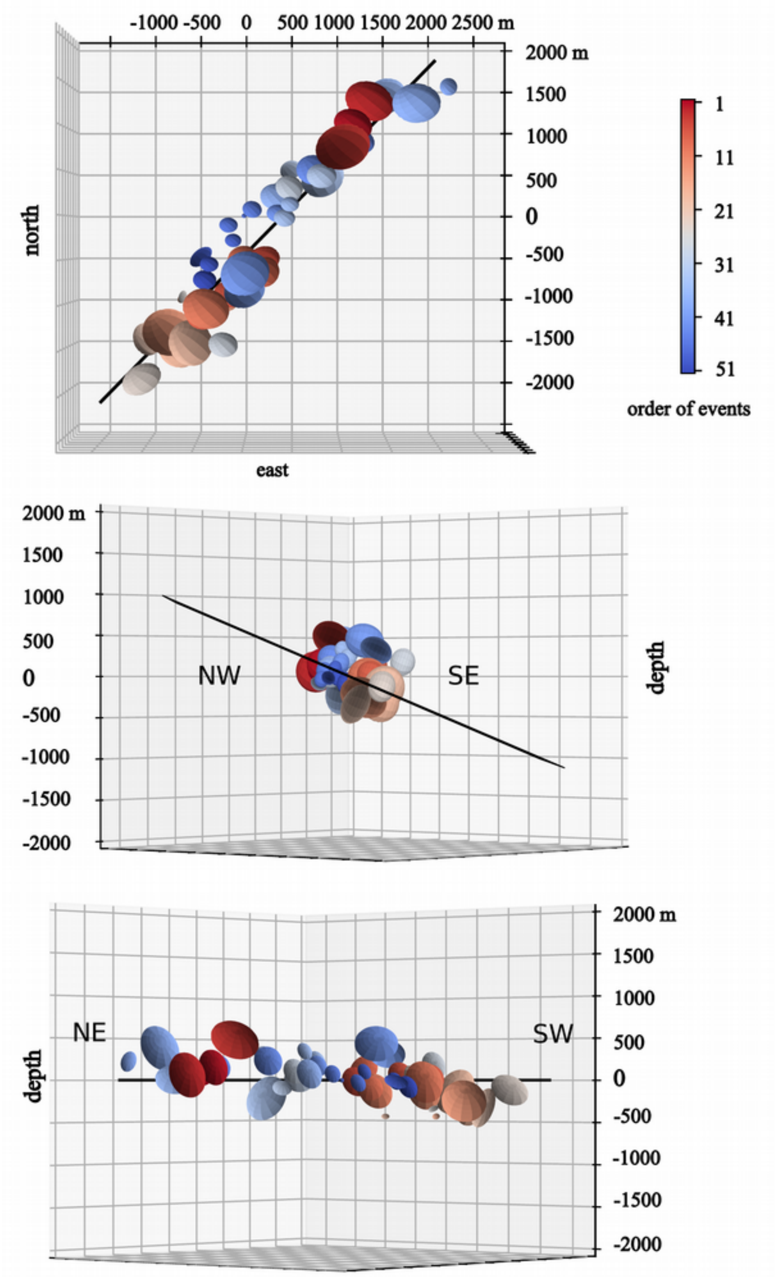

Supplementary Fig. 8: Uncertainties of the relocation based on waveform cross-correlation relocation. Uncertainty ellipsoids (1 σ) are plotted for relocated hypocenters, using colors to denote the temporal evolution (see color bar, reflecting the events chronological order).

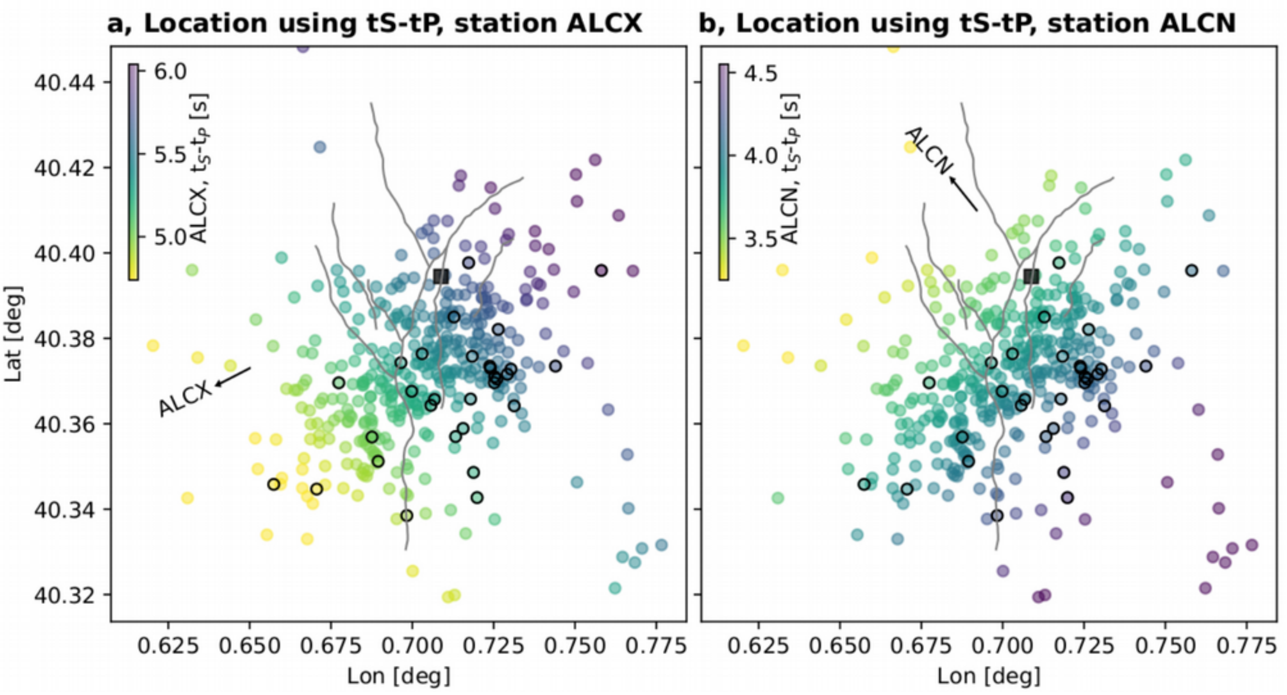

Supplementary Fig. 9: Locations based on tS-tP delays

The figures shows earthquake locations coloured according to tS-tP delays **a,** at station ALCX and **b,** at station ALCN. Black circles denote those events used in the waveform correlation location (a), for which S-P time estimates are available.

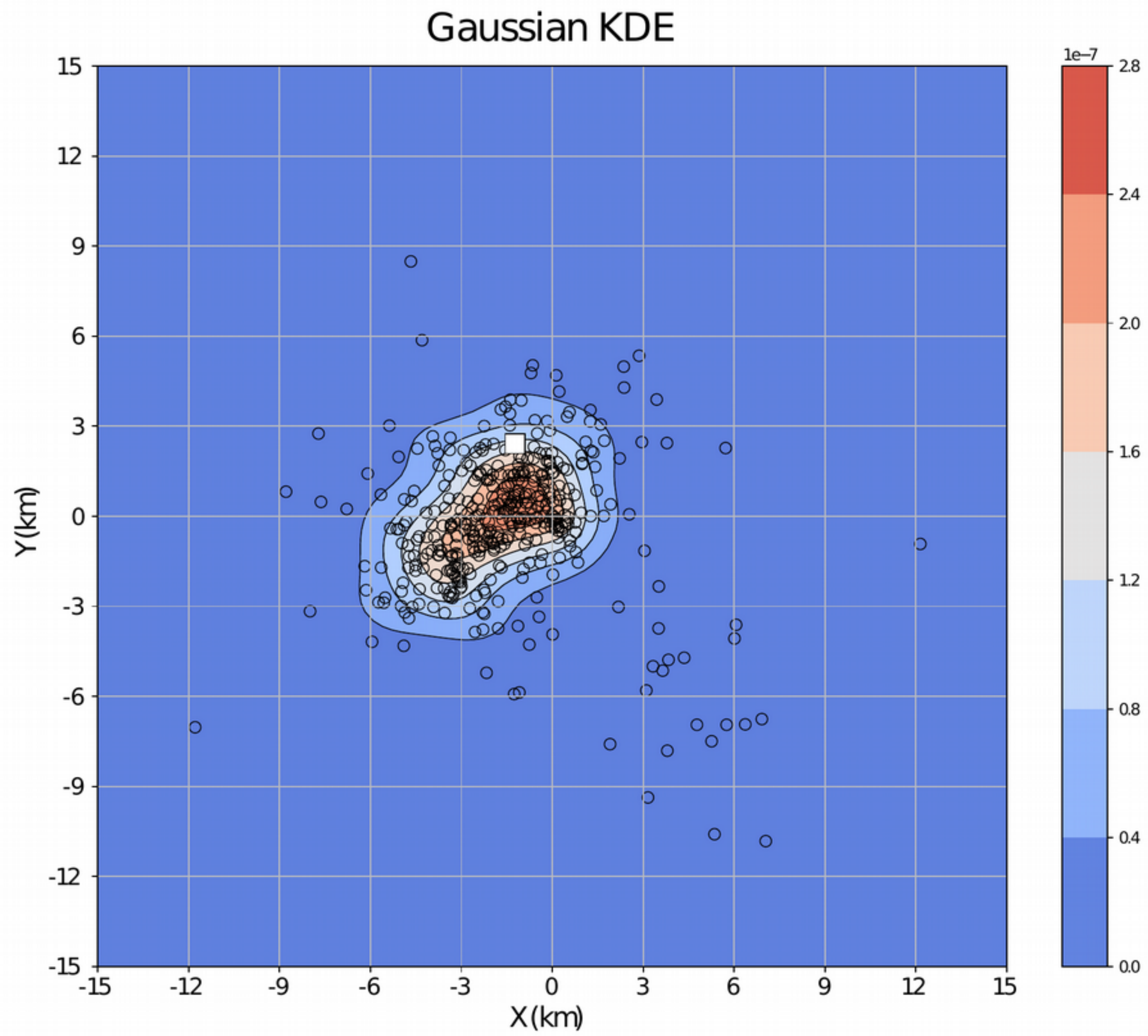

Supplementary Fig. 10: Stability of location results for velocity model perturbations

Gaussian Kernel Density Estimation (KDE) of the seismicity relocated 15 times, after perturbing the velocity model within the range 4.0–6.0 km/s for the V_P, (with velocity steps of 0.5 km/s) and the V_P/V_S ratio within the range 1.67–1.79 (with steps of 0.06). The circles represent the event-wise mean for each seismic event (relocated 15 times). The color scale from cyan to the red highlights the region with highest sample density (the total number of samples of 408 events x 15 iterations). The resolved high density region fits well the seismicity distribution, as an indication of the stability of the location results against velocity variation. This is also confirmed by a relatively small event-wise standard deviation which is of the order of 0.5 km for most of the events.

Supplementary Note 7, Moment tensor inversion

We report here the full table of moment tensor (MT) inversion results (Supplementary Table 2). Solutions are reported for 11 largest events in the Castor sequence, for which we can obtain robust results. Suppl. Table 2 report selected source parameters with their uncertainties and quantify some minor differences when assuming the three crustal models G, I and V to model synthetic seismograms and spectra. Moment tensor solutions are classified into A, B, and C quality, depending on their magnitudes (quality A corresponds to Mw 4.0-4.1, quality B to Mw 3.3-3.9, quality C to Mw 3.0-3.2). Solutions C are less stable, due to the weak magnitudes and acceptable signal-to-noise ratio at a lower number of close distance seismic stations. Suppl. Figs. 11-13 provide a complete overview on data fit for a selected earthquake (October 2, 2013, 23:06 UTC), complementing Fig. 3.

		Model G					Model I					Model V				
Event	Qual.	Mw	mean	std	min	max	Mw	mean	std	min	max	Mw	mean	std	min	max
Castor_20130924_002150	B	3.54	3.46	0.05	3.41	3.50	3.68	3.69	0.04	3.65	3.73	3.46	3.48	0.09	3.39	3.57
Castor_20130929_163623	B	3.41	3.43	0.05	3.38	3.48	3.70	3.68	0.04	3.64	3.72	3.36	3.38	0.08	3.30	3.46
Castor_20130929_211505	C	3.22	3.25	0.07	3.18	3.32	3.46	3.44	0.05	3.38	3.49	3.23	3.23	0.06	3.17	3.29
Castor_20130929_212316	C	3.02	3.11	0.07	3.05	3.18	3.38	3.33	0.06	3.27	3.39	3.05	3.10	0.06	3.04	3.16
Castor_20130929_221548	B	3.62	3.65	0.05	3.60	3.70	3.86	3.87	0.04	3.83	3.91	3.66	3.67	0.05	3.62	3.72
Castor_20130930_022116	B	3.91	3.85	0.04	3.81	3.89	4.08	4.07	0.03	4.04	4.10	3.89	3.85	0.04	3.81	3.89
Castor_20131001_033244	A	4.07	4.10	0.05	4.05	4.15	4.34	4.32	0.03	4.29	4.35	4.10	4.10	0.07	0.07	4.17
Castor_20131002_230649	A	4.11	4.05	0.05	4.00	4.10	4.27	4.30	0.03	4.27	4.33	4.00	3.96	0.08	3.88	4.04
Castor_20131002_232929	A	3.96	3.96	0.04	3.92	4.00	4.19	4.18	0.04	4.14	4.22	3.94	3.95	0.06	3.89	4.01
Castor_20131004_084948	B	3.66	3.65	0.04	3.61	3.69	3.87	3.88	0.04	3.84	3.91	3.62	3.67	0.06	3.61	3.73
Castor_20131004_095519	B	3.45	3.46	0.05	3.41	3.51	3.68	3.69	0.04	3.65	3.73	3.42	3.47	0.07	3.40	3.54
Maximum		4.11	4.10				4.34	4.32				4.10	4.10			
Event		Depth	mean	std	min	max	Depth	mean	std	min	max	Depth	mean	std	min	max
Castor_20130924_002150	B	1.6	1.9	1.5	0.4	3.4	2.0	2.9	2.6	0.3	5.5	4.3	7.1	4.3	2.8	11.4
Castor_20130929_163623	B	1.8	2.2	2.0	0.2	4.2	1.8	2.9	2.7	0.2	5.6	4.2	6.4	4.2	2.2	10.6
Castor_20130929_211505	C	1.7	6.7	5.0	1.7	11.7	2.1	5.0	4.1	0.9	9.1	8.7	10.3	3.0	7.3	13.3
Castor_20130929_212316	C	1.1	6.1	5.0	1.1	11.1	3.6	6.9	4.4	2.5	11.3	3.6	9.8	4.3	5.5	14.1
Castor_20130929_221548	B	1.9	4.0	3.4	0.6	7.4	2.6	4.3	3.1	1.2	7.4	8.0	9.2	3.7	5.5	12.9
Castor_20130930_022116	B	1.5	3.8	2.7	1.1	6.5	2.8	4.3	2.5	1.8	6.8	8.1	7.8	3.3	4.5	11.1
Castor_20131001_033244	A	2.3	2.2	0.8	1.4	3.0	2.1	2.4	1.0	1.4	3.4	3.5	6.3	3.3	3.0	9.6
Castor_20131002_230649	A	1.6	1.9	0.8	1.1	2.7	2.2	2.2	1.1	1.1	3.3	3.9	3.7	2.8	0.9	6.5
Castor_20131002_232929	A	2.9	2.3	0.7	1.6	3.0	3.0	2.7	1.3	1.4	4.0	4.3	5.3	2.8	2.5	8.1
Castor_20131004_084948	B	2.6	2.3	1.1	1.2	3.4	2.9	3.1	2.3	0.8	5.4	4.4	6.3	3.5	4.0	9.8
Castor_20131004_095519	B	3.1	3.1	2.5	0.6	5.6	2.8	3.0	1.5	1.5	4.5	3.8	6.2	3.1	3.1	9.3
Mean (Quality A+B)		2.14	2.63	1.72			2.47	3.09				4.94	6.48			
Event		Strike	mean	std	min	max	Strike	mean	std	min	max	Strike	mean	std	min	max
Castor_20130924_002150	B	45	67	41	26	108	44	66	39	27	105	52	81	19	62	100
Castor_20130929_163623	B	43	59	43	16	102	43	47	10	37	57	45	64	43	21	107
Castor_20130929_211505	C	25	55	51	4	106	34	85	80	5	165	239	205	67	138	272
Castor_20130929_212316	C	43	29	89	-60	118	49	67	58	9	125	44	79	70	9	149
Castor_20130929_221548	B	38	51	40	11	91	37	59	49	10	108	38	89	72	17	161
Castor_20130930_022116	B	41	48	30	18	78	40	47	35	12	82	45	76	78	-2	154
Castor_20131001_033244	A	41	57	46	11	103	41	52	31	21	83	49	82	73	9	155
Castor_20131002_230649	A	44	59	53	6	112	42	43	13	30	56	43	81	62	19	143
Castor_20131002_232929	A	43	50	41	9	91	38	65	55	10	120	39	78	71	7	149
Castor_20131004_084948	B	47	56	31	25	87	43	59	38	21	97	48	69	55	14	124
Castor_20131004_095519	B	39	45	22	23	67	39	52	36	16	88	42	72	65	7	137
Mean (Quality A+B)		42	55	39			41	54	34			45	77	60		
St. Dev. (Quality A+B)		3	7				2	8				5	8			
Event		Dip	mean	std	min	max	Dip	mean	std	min	max	Dip	mean	std	min	max
Castor_20130924_002150	B	34	55	13	42	68	59	62	12	50	74	44	64	12	52	76
Castor_20130929_163623	B	54	53	14	39	67	41	49	10	39	59	62	61	13	48	74
Castor_20130929_211505	C	66	72	12	60	84	73	67	13	54	80	45	61	19	42	80
Castor_20130929_212316	C	79	74	13	61	87	48	54	15	39	69	81	57	16	41	73
Castor_20130929_221548	B	46	46	12	34	58	54	54	12	42	66	60	58	13	45	71
Castor_20130930_022116	B	31	45	12	33	57	47	52	10	42	62	49	55	15	40	70
Castor_20131001_033244	A	57	51	16	35	67	52	57	13	44	70	53	62	14	48	76
Castor_20131002_230649	A	41	50	15	35	65	50	47	11	36	58	64	70	13	57	83
Castor_20131002_232929	A	58	59	13	46	72	57	64	12	52	76	79	70	15	55	85
Castor_20131004_084948	B	53	56	11	45	67	58	63	12	51	75	65	64	12	52	76
Castor_20131004_095519	B	62	54	12	42	66	63	60	12	48	72	58	65	13	52	78
Mean (Quality A+B)		48	52	13			53	56	12			59	63	13		
St. Dev. (Quality A+B)		11	5				7	6				10	5			
Event		Rake	mean	std	min	max	Rake	mean	std	min	max	Rake	mean	std	min	max
Castor_20130924_002150	B	4	16	73	-57	89	-4	10	74	-64	84	7	-36	85	-121	49
Castor_20130929_163623	B	1	0	52	-52	52	-1	3	18	-15	21	3	-11	70	-81	59
Castor_20130929_211505	C	-42	-32	46	-78	14	-27	-37	77	-114	40	8	-51	79	-130	28
Castor_20130929_212316	C	-30	-20	130	-150	110	6	-15	59	-74	44	-6	-17	68	-85	51
Castor_20130929_221548	B	-18	-23	51	-74	28	-12	-11	72	-83	61	-16	-45	93	-138	48
Castor_20130930_022116	B	-9	-23	39	-62	16	-15	-14	-37	23	-51	-14	-19	69	-88	50
Castor_20131001_033244	A	0	0	46	-46	46	0	12	51	-39	63	9	-27	84	-111	57
Castor_20131002_230649	A	0	-9	49	-58	40	0	-1	14	-15	13	10	-24	92	-116	68
Castor_20131002_232929	A	10	6	49	-43	55	12	28	72	-44	100	25	-20	75	-95	55
Castor_20131004_084948	B	4	7	52	-45	59	-3	3	63	-60	66	2	-22	73	-95	51
Castor_20131004_095519	B	-3	4	39	-35	43	9	16	64	-48	80	11	-25	77	-102	52
Mean (Quality A)		-1	-2	50			-2	5	43			4	-25	80		
St. Dev. (Quality A)		8	13				9	13				13	10			

Supplementary Tab. 2: Summary of MT inversion results.

We obtain MT solutions for 11 earthquakes with quality A to C (magnitude-based), using models G (purple), I (blue) and V (red), reporting magnitude (Mw), depth (km), strike, dip and rake (deg) for the best (using all data) and mean (based on data bootstrap) solutions, with their uncertainties, as well as average solutions for the whole sequence.

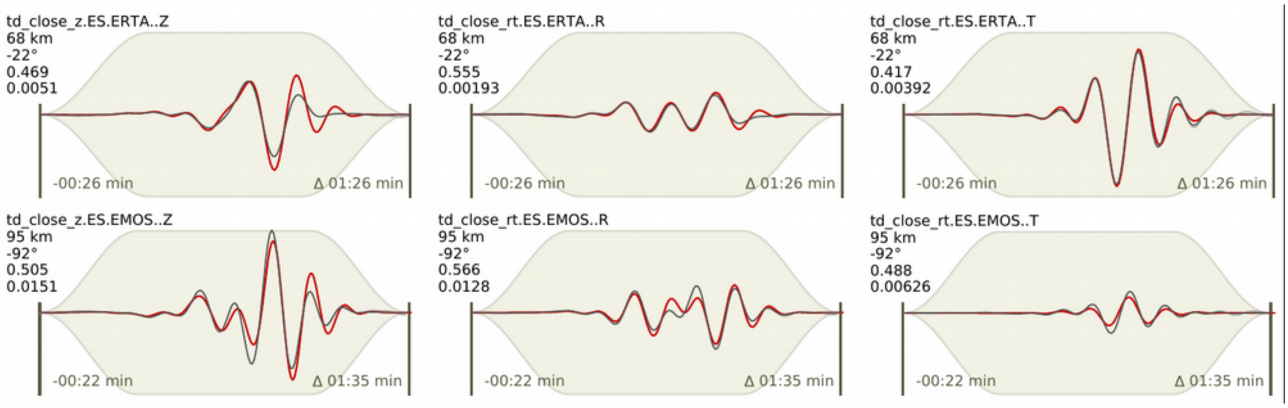

Supplementary Fig. 11: Fit of full waveforms in time domain, for the October 2, 2013, UTC 23:06 Castor earthquake, for the closest broadband stations (below 100 km).

Observed (black lines) and synthetics (red lines) displacement seismograms (bandpass 0.02-0.05 Hz) are fitted for the vertical (A), radial (B) and transversal (C) components (fitting full waveforms in the time domain, synthetic and observed waveform amplitudes are comparable). Station information is provided on the top left part of each waveform (as in Fig. 3).

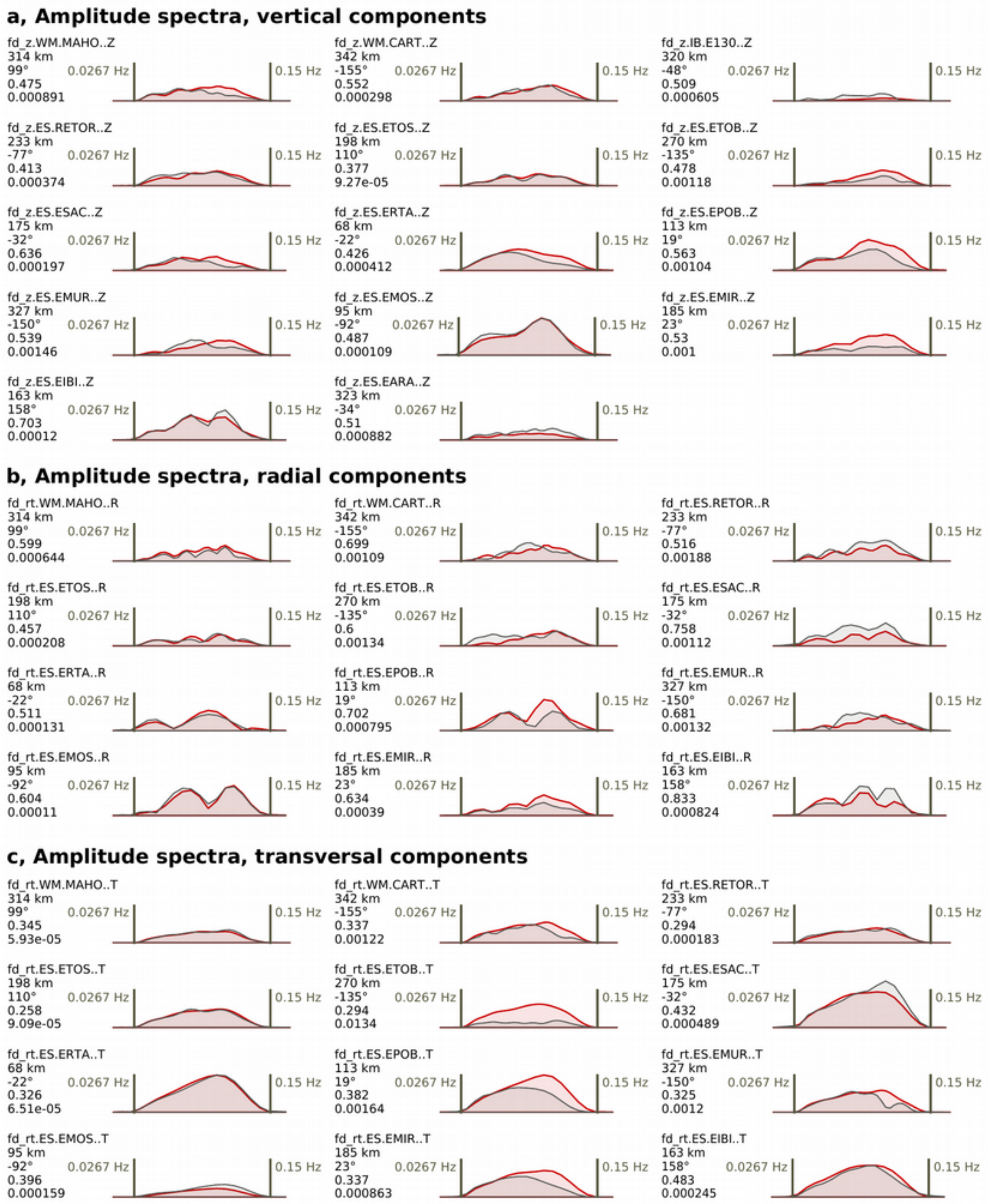

Supplementary Fig. 12: Fit of full waveform amplitude spectra, for the October 2, 2013, UTC 23:06 Castor earthquake.

Observed (black lines) and synthetics (red lines) displacement spectra are fitted **a,** for the vertical, **b,** radial and **c,** transversal components (the frequency band is indicated in the vertical bars). Station information is provided on the top left part of each waveform (as in Fig. 3).

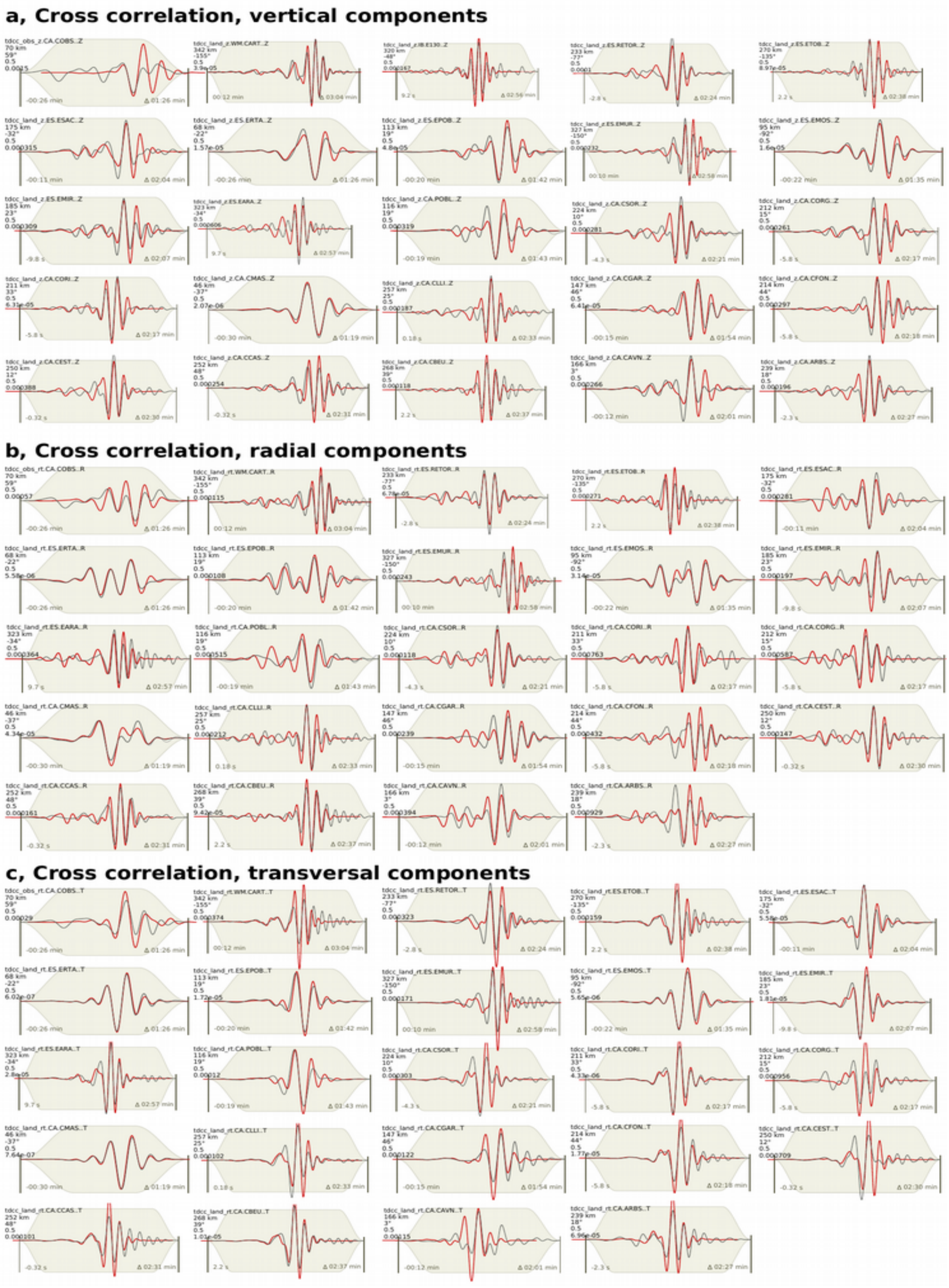

Supplementary Fig. 13: Fit of full waveforms by cross correlations, for the October 2, 2013, UTC 23:06 Castor earthquake.

Observed (black lines) and synthetics (red lines) displacement seismograms (bandpass 0.02-0.05 Hz) are fitted for **a,** the vertical, **b,** radial and **c,** transversal components (fitting by cross correlation, synthetic and observed waveform amplitudes may differ). Station information is provided on the top left part of each waveform (as in Fig. 3).

Supplementary Note 8, Depth estimation using depth-phases

For this analysis Green's functions are computed using a reflectivity approach (QSEIS[15]), and considering a local crustal model at the source (here model G[10]), a local crustal model at the array location (here, extracted from the CRUST2.0 database[16]) and a common mantle model (AK135[17]). The Green functions with this setup are computed for bodywaves only, assuming a range of possible source depth, and we look at P pulses and consequent depth phases. Finally, since the focal mechanism is known (from the moment tensor inversion), we can compute synthetic beams for the proper mechanism and different depths and compare them to the observed beam.

The algorithm used is open source (https://github.com/HerrMuellerluedenscheid/abedeto). The method is suited for shallow seismicity and it has been successfully applied in recent applications for natural and induced seismicity and explosion signals[18,19,20,21].

Suppl. Fig. 14 shows the comparison of observed and modeled beams at the GERES array, similar as in Fig. 4, for the three largest earthquakes of the Castor sequence.

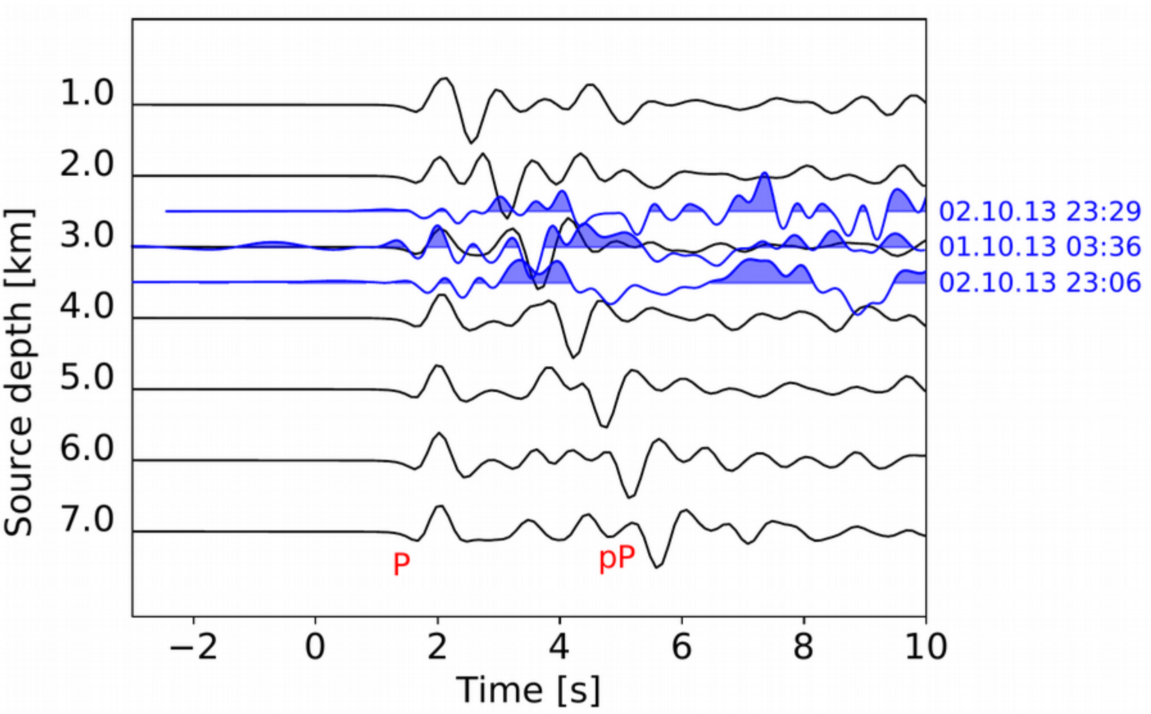

Supplementary Fig. 14: Comparison of observed beams for the three largest earthquake at GERES, compared to synthetics for different source depths.

Observed beams are in blue, and the name of the event is listed, synthetic for the reference mechanism (Fig. 5) in black. Model G is used. Red P and pP labels mark the arrival time at 7 km depth, where they are well separated.

Supplementary Note 9, Rupture directivity

Results of the rupture directivity analysis are reported in Supplementary Table 3. The stability of the apparent source time function duration (ASTF) estimations is illustrated in Suppl. Fig. 15, where we compare results using different earthquakes as empirical Green's functions.

	Earthquake 1 (2013-10-01 03:32)	Earthquake 2 (2013-10-02 23:06)
Directivity (°)	15 ± 15	17 ± 19
Rupture asymmetry (%)	66 ± 6	75 ± 7
Rupture length (km)	1.0 ± 0.6	1.2 ± 0.6
Rupture velocity (km/s)	2.7 ± 3.5	2.7 ± 2.3
Total rupture time (s)	0.50 ± 0.14	0.47 ± 0.13
Rise time (s)	0.25	0.15

Supplementary Tab. 3: Results of the EGF analysis.

The table reports directivity direction, percentage of rupture asymmetry (100% means purely unilateral rupture, 50% purely bilateral rupture, rupture length, rupture velocity, rutpure time and rise time, and their uncertainties (the rise time was here fixed), for the two target earthquakes on October 1, 2013, 03:32 UTC and October 2, 2013, 23:06.

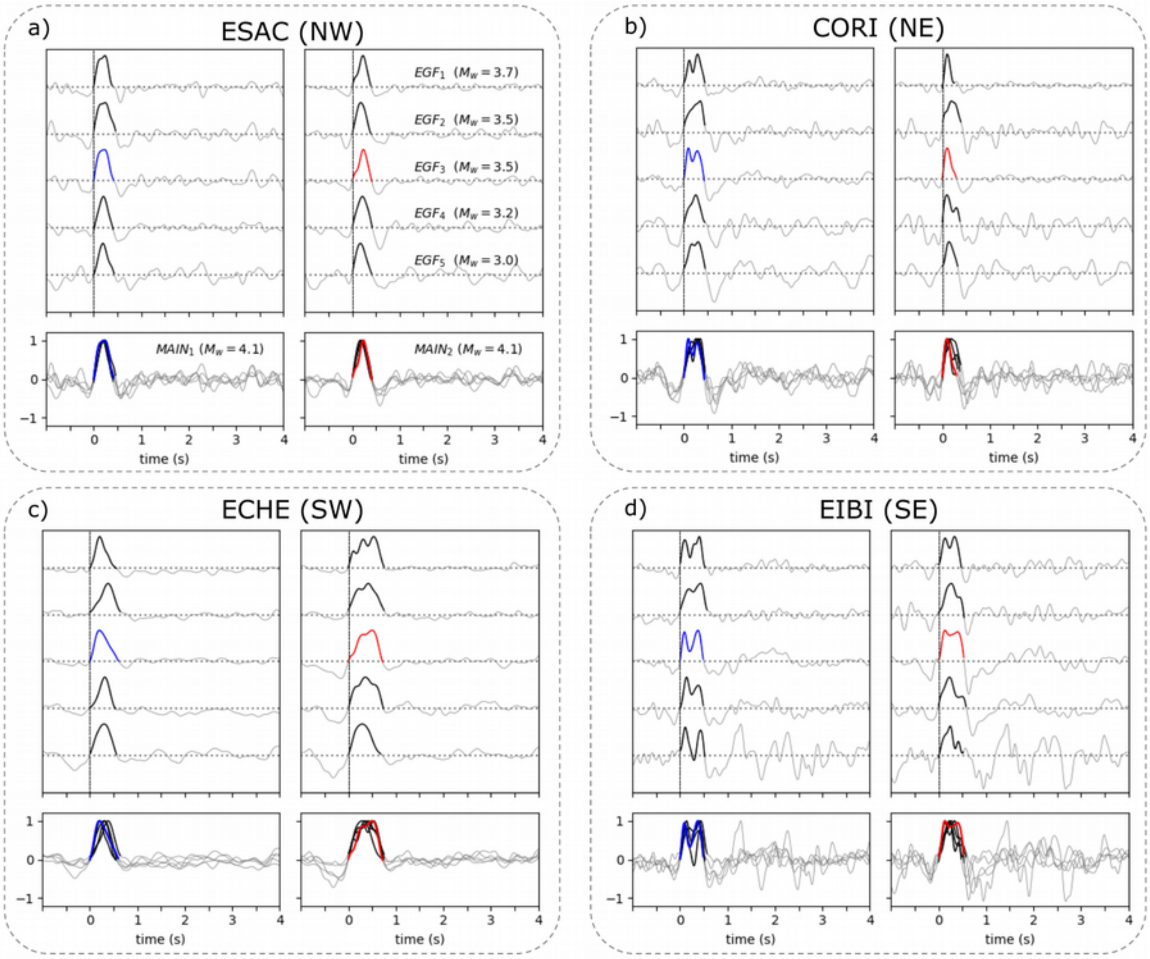

Supplementary Fig. 15: Stability of Apparent Source Time Function (ASTF) estimation. Comparison of results using different earthquakes as Empirical Green's Functions (EGFs) for the October 1, 2013, 1 (first column in each panel) and October 2, 2013, (second column) M_w 4.1 earthquakes. ASTFs are obtained using different EGFs (EGF1 October 4, 2013, 08:49, EGF2 September 24, 2013, 00:21, EGF3: October 4, 2013, 09:55, EGF4: September 29, 2013, 21:15, EGF5: October 4, 2013, 20:02), for seismic stations located NW (**a,** station ESAC), NE (**b,** CORI), SW (**c,** ECHE) and SE (**d,** EIBI) of the focal region. ASTFs are labeled (a) for each EGF, sorted by decreasing seismic moment. The bottom row in each panel shows the overlapped normalized ASTFs. Gray traces show the deconvolved functions from S waves through spectral division and the resulting ASTF is highlighted with a black line. We finally select the October 4, 2013, 09:55 as EGF (blue and red traces are used for P and S phases repsectively, for the ASTF obtained for the selected EGF and in bottom panels).

Supplementary Note 10, Waveform similarity of relocated events

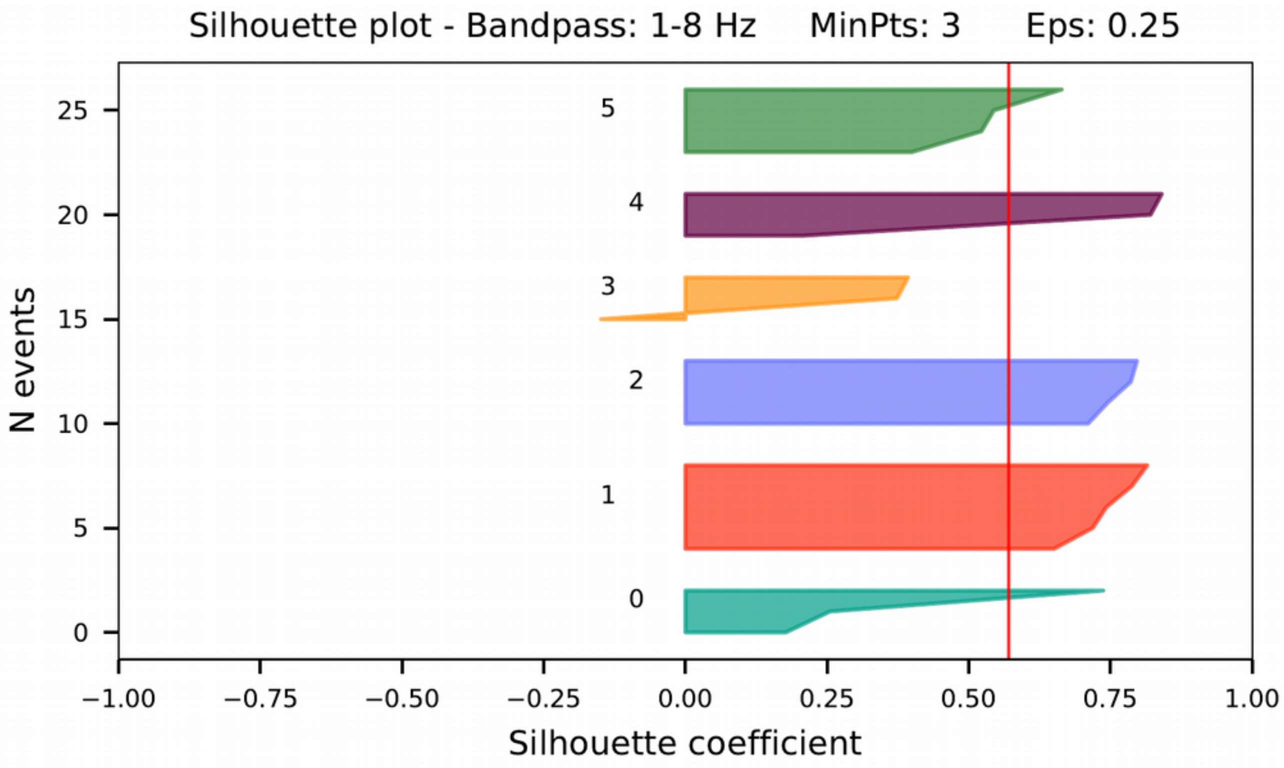

Supplementary Fig. 16: Silhouette plot for the waveform based clustering results.
The silhouette plot shows that the clusters identified in the Castor sequence are in general well separated from each other. The silhouette coefficient is a measure for the similarity of an event in regard to other events in its cluster compared to the similarity to events in other clusters. Here, the silhouette coefficients of the events of one cluster form a polygon with rather rectangular shapes indicating more homogeneous clusters. A negative value indicates that an event might be better assigned to another cluster. Clusters are colored according to Fig. 7.

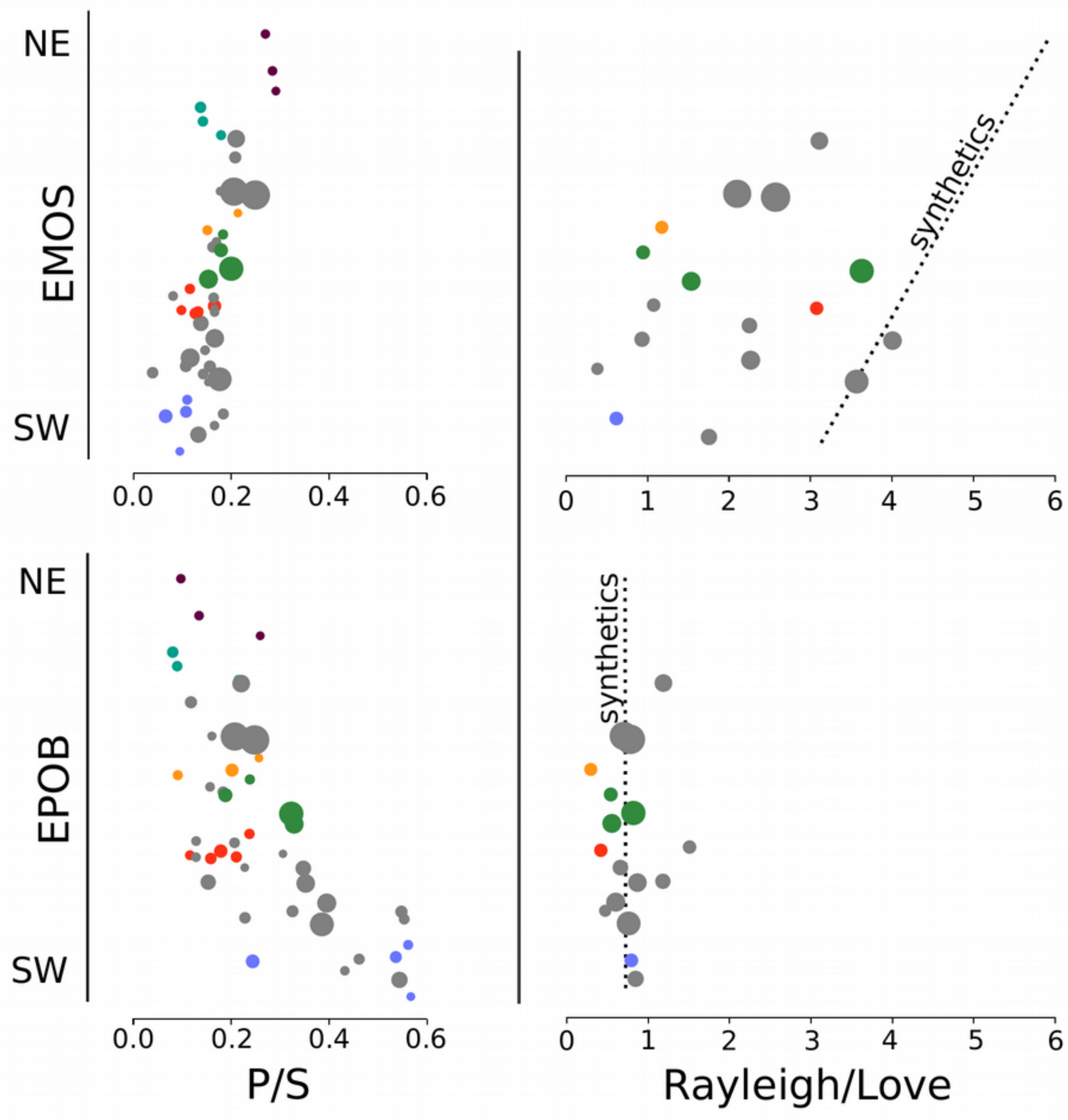

Supplementary Fig. 17: Waveform based clustering results: modeling of P/S and Rayleigh/Love ratios along a NE-SW profile.

The ratios P/S at station EPOB and R/L at station EMOS show smooth trends along the NE-SW profile CD (Fig. 2). Clusters are colored according to Fig. 7.

Supplementary References

1. Foulger, G. R., Wilson, M. P., Gluyas, J. G., Julian, B. R. & Davies, R. J. Global review of human-induced earthquakes. *Earth-Science Reviews* **178**, 438-514, https://doi.org/10.1016/j.earscirev.2017.07.008 (2018).

2. Cesca, S., et al. Source modelling of the *M*5–6 Emilia-Romagna, Italy, earthquakes (2012 May 20–29), *Geophys. J. Int.*, **193**, 3, 1658–1672, https://doi.org/10.1093/gji/ggt069 (2013).

3. Grigoli, F., et al. The November 2017 *M*w 5.5 Pohang earthquake: A possible case of induced seismicity in South Korea. *Science* **360**, 6392, 1003-1006, https://doi.org/10.1126/science.aat2010 (2018).

4. Instituto Geografico Nacional, Spain. Spanish Digital Seismic Network, International Federation of Digital Seismograph Networks, https://doi.org/10.7914/SN/ES (1999).

5. Institut Cartogràfic I Geològic De Catalunya. Catalan Seismic Network. International Federation of Digital Seismograph Networks, https://doi.org/10.7914/SN/CA (1984).

6. San Fernando Royal Naval Observatory (ROA), Universidad Complutense De Madrid (UCM), Helmholtz-Zentrum Potsdam Deutsches GeoForschungsZentrum (GFZ), Universidade De Evora (UEVORA, Portugal) & Institute Scientifique Of Rabat (ISRABAT, Morocco). The Western Mediterranean BB seismic Network. Deutsches GeoForschungsZentrum GFZ. https://doi.org/10.14470/JZ581150 (1996).

7. Institute Earth Sciences "Jaume Almera" CSIC (ICTJA Spain), IberArray. International Federation of Digital Seismograph Networks, https://doi.org/10.7914/SN/IB (2007).

8. Federal Institute for Geosciences and Natural Resources (BGR), German Regional Seismic Network (GRSN), https://doi.org/10.25928/mbx6-hr74 (1976).

9. Cesca, S., et al. The 2013 september-october seismic sequence offshore spain: a case of seismicity triggered by gas injection? *Geophys. J. Int.* **198**, 941–953, https://doi.org/10.1093/gji/ggu172 (2014).

10. Gaite, B., Ugalde, A., Villaseñor, A. & Blanch, E. Improving the location of induced earthquakes associated with an underground gas storage in the Gulf of Valencia (Spain), *Phys. Earth Planet. Int.*, **254**, 46-59, https://doi.org/10.1016/j.pepi.2016.03.006 (2016).

11. Mezcua, J. & Martinez Solares, J. M. Sismicidad del Area Ibero-Mogrebi, Instituto Geográfico Nacional Publ., Madrid, 189 pp. (1983).

12. Vidal, N., Gallart, J., Dañobeitia, J. J. A deep seismic crustal transect from the NE Iberian Peninsula to the western Mediterranean. *J. Geophys. Res.* **103** (B6), 12381–12396. http://dx.doi.org/10.1029/98JB00076 (1998).

13. González, Á. The Spanish National Earthquake Catalogue: Evolution, precision and completeness, *J. Seismol.* **21**, 3, 435-471 (2017).

14. Wiemer, S. & Wyss, M. Minimum magnitude of completeness in earthquake catalogs: examples from Alaska, the Western United States & Japan, *Bull. seism. Soc. Am.* **90**, 859–869. https://doi.org/10.1785/0119990114 (2000).

15. Wang, R., A simple orthonormalization method for the stable and efficient computation of Green's functions, *Bull. Seismol. Soc. Am.*, **89**, 733-741 (1999).

16. Bassin, C., Laske, G. & Masters, G. The Current Limits of Resolution for Surface Wave Tomography in North America, *EOS Trans AGU*, **81**, F897 (2000).

17. Kennett B.L.N., Engdahl E.R. & Buland R. Constraints on seismic velocities in the earth from travel times, *Geophys. J. Int.*, **122**, 108-124 (1995).

18, Negi, S. S., Paul, A., Cesca, S., Kamal, Kriegerowski, M., Mahesh, P. & Gupta, S. Crustal velocity structure and earthquake processes of Garhwal-Kumaun Himalaya: Constraints from regional waveform inversion and array beam modeling. *Tectonophysics*, **712-713**, 45-63, https://doi.org/10.1016/j.tecto.2017.05.007 (2017).

19. Gaebler, P., Ceranna, L., Nooshiri, N., Barth, A., Cesca, S., Frei, M., Grünberg, I., Hartmann, G., Koch, K., Pilger, C., Ross, J. O. & Dahm, T. 2019, A multi-technology analysis of the 2017 North Korean nuclear test, *Solid Earth*, **10**, 59-78, https://doi.org/10.5194/se-10-59-2019

20. Mouslopoulou, V., Bocchini, G. M., Cesca, S., Saltogianni, V., Bedford, J., Petersen, G., Gianniou, M. & Oncken, O. Earthquake Swarms, Slow Slip and Fault Interactions at the Western-End of the Hellenic Subduction System Precede the Mw6.9 Zakynthos Earthquake, Greece, *Geochemistry Geophysics Geosystems (G3)*, **21**, 12, https://doi.org/10.1029/2020GC009243 (2020).

21. Büyükakpınar, P., Cesca, S., Hainzl, S., Jamalreyhani, M., Heimann, S., & Dahm, T. Reservoir-triggered earthquakes around the Atatürk Dam (southeastern Turkey), *Frontiers in Earth Science*, https://doi.org/10.3389/feart.2021.663385 (2021).
