# Supplementary material for: Seismicity at the Castor gas reservoir driven by pore pressure diffusion and asperities loading
Source: Nat Commun. 2021 Aug 10;12:4783. doi: 10.1038/s41467-021-24949-1 (PMC8355105; doi:10.1038/s41467-021-24949-1)
Supplement: Supplementary file 3 — Description of Additional Supplementary Files [file 41467_2021_24949_MOESM3_ESM.pdf]

Description of Additional Supplementary Files

File name: Supplementary Data 1
Description: Template matching catalog, including 3,437 events .

File name: Supplementary Data 2
Description: Relative location catalog based on waveform correlation, including 51 events .

File name: Supplementary Data 3
Description: Relocated catalog based on tS-tP and distance geometry technique, including 408 events.
